# Length of hospitalization and mortality for bleeding during treatment with warfarin, dabigatran, or rivaroxaban

**DOI:** 10.1371/journal.pone.0193912

**Published:** 2018-03-28

**Authors:** Blake Charlton, Gboyega Adeboyeje, John J. Barron, Deborah Grady, Jaekyu Shin, Rita F. Redberg

**Affiliations:** 1 Department of Medicine, University of California San Francisco, San Francisco, CA, United States of America; 2 HealthCore, Inc, Wilmington, DE, United States of America; 3 Department of Clinical Pharmacy, School of Pharmacy, University of California San Francisco, CA, San Francisco, CA, United States of America; National Yang-Ming University, TAIWAN

## Abstract

**Background:**

Different outcomes among patients hospitalized for bleeding after starting anticoagulation could influence choice of anticoagulant. We compared length of hospitalization, proportion of Intensive Care Unit (ICU) admissions, ICU length of stay, and 30- and 90-day mortality for adults with atrial fibrillation hospitalized for bleeding after starting warfarin, dabigatran, or rivaroxaban.

**Methods:**

An US commercial database of 38 million members from 1 November 2010 to 31 March 2014 was used to examine adults with atrial fibrillation hospitalized for bleeding after starting warfarin (2,446), dabigatran (442), or rivaroxaban (256). Outcomes included difference in mean total length of hospitalization, proportion of ICU admissions, mean length of ICU stay, and all-cause 30- and 90-day mortality.

**Results:**

Warfarin users were older and had more comorbidities. Multivariable regression modeling with propensity score weighting showed warfarin users were hospitalized 2.0 days longer (95% CI 1.8–2.3; p < 0.001) than dabigatran users and 2.6 days longer (95% CI 2.4–2.9; p < 0.001) than rivaroxaban users. Dabigatran users were hospitalized 0.6 days longer (95% CI 0.2–1.0; p = 0.001) than rivaroxaban users. There were no differences in the proportion of ICU admissions. Among ICU admissions, warfarin users stayed 3.0 days (95% CI 1.9–3.9; p < 0.001) longer than dabigatran users and 2.4 days longer (95% CI 0.9–3.7; p = 0.003) than rivaroxaban users. There was no difference in ICU stay between dabigatran and rivaroxaban users. There were no differences in 30- and 90-day all-cause mortality.

**Conclusions:**

Rivaroxaban and dabigatran were associated with shorter hospitalizations; however, there were no differences in 30- and 90-day mortality. These findings suggest bleeding associated with the newer agents is not more dangerous than bleeding associated with warfarin.

## Introduction

The development of Non-vitamin K Oral Anticoagulants (NOACs) has provided an alternative to warfarin for stroke prophylaxis in atrial fibrillation. Two widely used NOACs are dabigatran, a direct thrombin inhibitor, and rivaroxaban, a factor Xa inhibitor. Two other factor Xa inhibitors, apixaban and edoxaban, are also FDA-approved. The effectiveness and safety—including incidence of bleeding—of each NOAC compared to warfarin have been studied in randomized controlled non-inferiority trials.[1]^,^[2]^,^[3]^,^[4]

One meta-analysis of these trials found that NOACs were associated with reduced incidence of stroke, intracranial hemorrhage, and mortality but similar incidence of bleeding compared to warfarin.[5] Two more recent analyses of clinical trials data found increased risk of gastrointestinal bleeding on NOACs.[6]^,^[7] Observational studies have demonstrated an increased risk of bleeding with dabigatran compared to warfarin,^9,10^ and similar risks of bleeding with rivaroxaban compared to warfarin.^11,12^

There has been little investigation to determine if NOAC-associated bleeding is more severe or complicated than warfarin-associated bleeding. Most research has focused on intracranial hemorrhage. One meta-analysis of trial data found NOACs to be associated with reduced mortality and intracranial hemorrhage but that, after adjusting for site of bleeding, there was no difference in incidence of fatal bleeding for any given bleeding site.[8] Observational studies have found intracranial hemorrhage during NOAC therapy was associated with better radiographic and functional outcomes.[9]^,^[10] A detailed determination of the relative complexity and severity of bleeding associated with these agents could help guide selection of oral anticoagulants and management of acutely bleeding patients.

This study seeks to investigate the complexity and severity of NOAC-associated bleeding by examining mean total length of hospitalization, proportion of patients admitted to the ICU, mean length of ICU stay, and all-cause 30- and 90-day mortality for adults with atrial fibrillation who were hospitalized for bleeding after starting warfarin, dabigatran, or rivaroxaban.

Because warfarin is more easily reversible than newer agents, we hypothesized that, outside of controlled trials, hospitalization for warfarin-associated bleeding may be associated with shorter stays, fewer ICU admissions, and lower mortality.

## Methods

### Study design

We conducted a retrospective cohort study of patients with atrial fibrillation who were hospitalized for bleeding after starting warfarin, dabigatran, or rivaroxaban using administrative claims data for outpatient, inpatient, and pharmacological treatments from the HealthCore Integrated Research Environment (HIRE) from 1 November 2010 through 31 March 2014.

The HIRE database includes adjudicated medical and pharmacy claims data for approximately 38 million members of large commercial health plans in 14 US states. Patients with missing pharmaceutical or medical claims were excluded. The database represents claims information from one of the largest commercially insured populations in the United States. To ensure completeness of claims submissions, we allowed a 3-month runout period, based on internal analyses indicating that virtually all claims become available within 3 months of an event.

**Fig 1:**
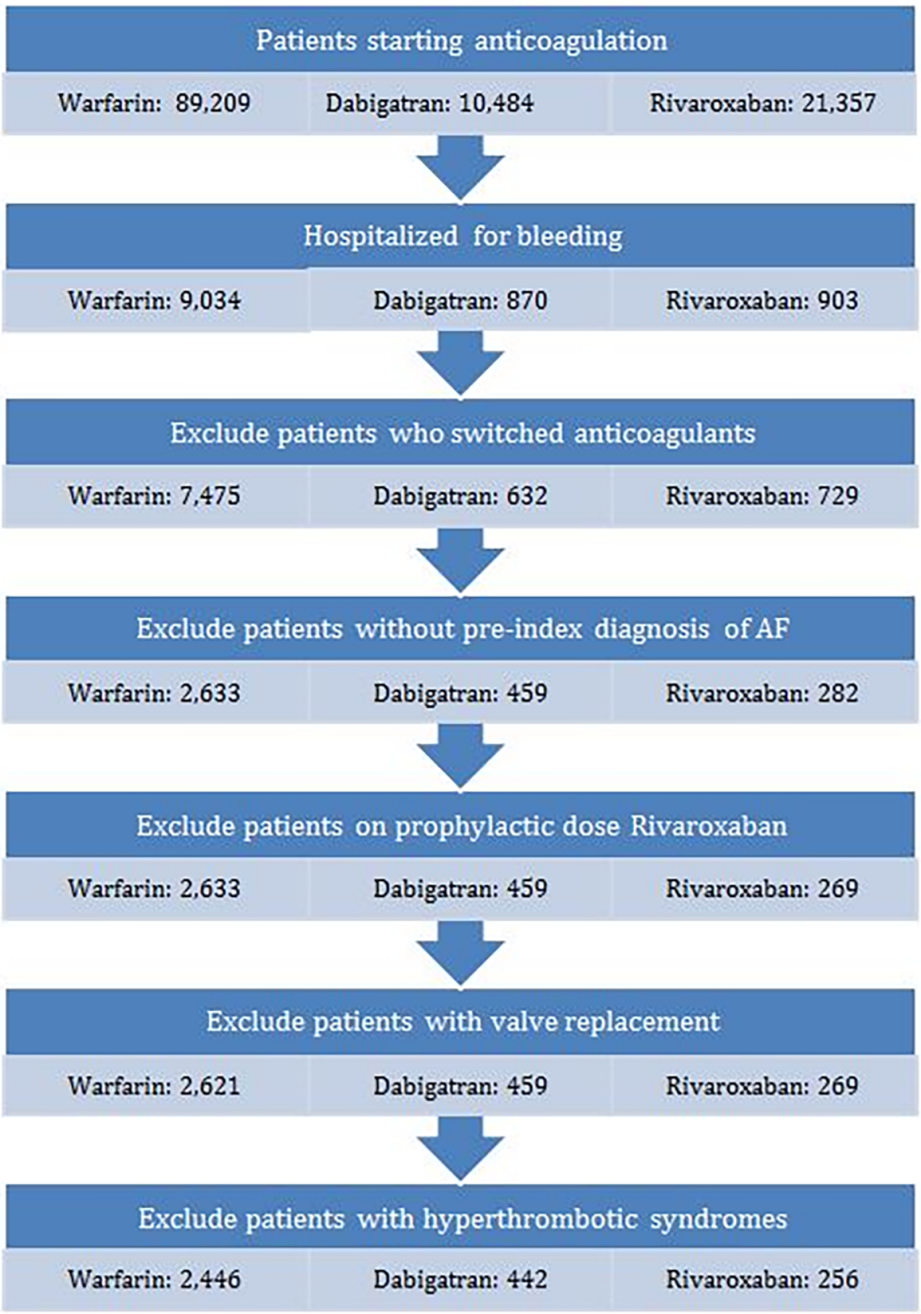
Cohort definition.

We included patients with atrial fibrillation who had filled a prescription for warfarin, dabigatran, or rivaroxaban and were subsequently hospitalized for bleeding between 1 November 2010 and 31 March 2014. To ensure the accuracy of the indication for anticoagulation, the diagnosis of atrial fibrillation was defined as the presence of ≥ 2 medical claims (inpatient, emergency department, and outpatient) with International Classification of Diseases, Ninth Revision (ICD9) Clinical Modification codes of 427.31 or 427.3 (if no 5^th^ digit) in the 6 month period before starting one of the index drugs. We used ICD-9 codes to identify hospitalization for bleeding based on previously described protocols with positive predictive values between 89 and 99%.[11]^,^[12]^,^[13]^,^[14]^,^[15]^,^[16] **(S1 Appendix)** To ensure that study participants were initiating anticoagulation, we excluded patients who had filled a prescription for any anticoagulant up to six months prior to starting the index drug. Patients who had not filled their anticoagulation prescriptions in the 60 days prior to hospitalization and those who had switched from one anticoagulant to another prior to their index hospitalization were excluded. We also excluded patients on rivaroxaban 10 mg (indicated only for venous thromboembolism prophylaxis), with a diagnosis of severe renal disease, cardiac valve replacement, mitral valve disorder, antiphospholipid antibody syndrome, protein C deficiency, protein S deficiency, factor V Leiden syndrome, antithrombin III deficiency, prothrombin 20210A mutation, nephrotic syndrome, paroxysmal nocturnal hemoglobinuria, polycythemia vera, or essential thrombocytosis. **(Fig 1, S1 Appendix**)

Researchers had access only to a de-identified data set. Strict measures were taken to preserve anonymity and confidentiality and to ensure full compliance with the 1996 Health Insurance Portability and Accountability Act; as such, the study was deemed exempt from review by the University of California San Francisco Institutional Review Board.

We identified death by discharge status within the HIRE database and by linking HIRE to the Social Security Administration Death Master File Index through 31 April 2014. Data linkage was confirmed using social security number and date of birth for more than 95% of subjects in HIRE.

### Outcomes

Outcomes included mean total length of hospital stay, proportion of patients admitted to the ICU, mean length of ICU stay, and all-cause 30- and 90-day mortality. Due to limitations of the data, we were unable to investigate transfusion of blood products or use of agents to reverse warfarin.

### Statistical analysis

We described baseline patient characteristics as means with standard deviations for normally distributed continuous variables and proportions for categorical variables. We compared baseline differences among patients in the three exposure groups using analysis of variance (ANOVA) for continuous variables and the Pearson χ^2^ test for categorical variables. Statistical analyses were conducted with SAS version 9.4 software (SAS Institute; Cary, NC).

We addressed confounding due to differences in baseline patient characteristics using multivariable Poisson regression models weighted by the inverse probability of treatment. This was done in two steps.

First, we specified a multinomial logistic regression model to determine the probability of starting warfarin, dabigatran, or rivaroxaban including age, sex, geographic region, chronic kidney disease, heart failure, coronary artery disease, myocardial infarction, ischemic stroke, transient ischemic attack, peripheral vascular disease, cancer, diabetes, hypertension, dyslipidemia, pericarditis, hyperthyroidism, dementia, gait abnormalities, dizziness, diabetic and alcoholic neuropathy, esophageal varices, major trauma, coagulation defect factors, and use of the following medications: antiplatelet agents, antiarrhythmics, diuretics, vasopressors, steroids, progestin, estrogen, proton pump inhibitors, amiodarone, ketoconazole, dronedarone, nonsteroidal anti-inflammatory drugs (NSAIDs), and cyclooxygenase 2 (COX2) inhibitors as predictors of treatment.[17] **(S1 Appendix)** Warfarin, the most commonly prescribed anticoagulant, was selected as the reference drug.

Second, since the three propensity scores add up to one and are complementary, we calculated the inverse weights for the treatment each patient received as the main variable to control for confounding. We assessed positivity by examining the distribution of the propensity scores for substantial overlap given the set of observed covariates. Balance achieved by the propensity scores was assessed by comparing the three treatment groups on their baseline covariates using ANOVA, which included the treatment groups weighted by the inverse probability of treatment received.[18] **(Table 1)** After inverse propensity score weighting, only the prevalence of Parkinson’s disease and cancer were significantly different among the three groups. Therefore, we included these covariates in the multivariable models weighted by the inverse probability of treatment received. Primary measures were analyzed as count data and proportions as appropriate.

**Table 1:** Patient characteristics, unadjusted and propensity score weighted p-values, by treatment group.

Characteristic	Warfarin (n = 2503)	Dabigatran (n = 461)	Rivaroxaban (n = 260)	Unadjusted p-value	Adjusted p-value
Age, mean (SD) yr	74.4 (11.0)	69.6 (12.6)	68.0 (12.5)	<0.001	0.436
Male sex	55.7	61.8	62.9	0.009	0.635
Medicare Supplemental	11.1	12.2	7.4	0.133	0.406
Medicare Advantage	59.4	31.7	30.5	<0.001	0.243
**Region**					
Missing	2.0	4.1	2.7	<0.001	0.526
Northeast	24.6	24.9	14.8		
Midwest	47.4	35.1	34.8		
West	11.0	14.5	18.8		
South	15.1	21.5	28.9		
**Indexes**					
Deyo-Charlson Comorbidity Index, mean, (SD)	3.1 (2.6)	2.0 (2.0)	2.3 (2.3)	<0.001	0.692
CHA2DS2-VASc, mean (SD)	4.6 (1.6)	3.8 (1.7)	3.8 (1.8)	<0.001	0.561
**Comorbid Illnesses**					
Ischemic stroke	11.9	10.4	11.7	0.668	0.403
Transient ischemic attack	3.7	4.5	3.1	0.607	0.215
Chronic kidney disease	22.6	9.8	12.9	<0.001	0.091
Myocardial Infarction	16.3	13.1	12.1	0.068	0.148
Heart Failure	46.8	35.3	35.9	<0.001	0.288
Cerebrovascular disease	20.2	16.5	15.6	0.058	0.266
Coronary artery disease	52.1	45.7	39.5	<0.001	0.383
Peripheral vascular disease	32.4	23.1	23.4	<0.001	0.080
Cancer	32.1	29.6	31.6	0.583	0.008
Osteoarthritis	29.0	21.9	23.0	0.002	0.281
Diabetes Mellitus	38.0	28.7	31.6	<0.001	0.316
Hypertension	94.2	93.2	91.0	0.106	0.865
Dyslipidemia	70.1	71.3	68.8	0.775	0.614
Pericarditis	1.1	1.6	1.2	0.667	0.426
Hyperthyroidism	2.0	2.0	0.4	0.165	0.880
Coagulation defect factors	7.0	2.9	3.9	0.002	0.192
Dementia	4.7	2.7	1.2	0.004	0.405
Parkinson’s disease	1.4	0.9	0.4	0.360	<0.001
Gait Abnormality	10.4	3.6	7.0	<0.001	0.496
Dizziness	12.8	14.3	12.1	0.654	0.479
Diabetic and alcoholic neuropathy	3.1	2.0	2.0	0.373	0.344
Esophageal varices	0.3	0.2	0.0	1.000	0.504
Major trauma	23.0	15.6	18.4	<0.001	0.116
**Pre-Index Medications**					
Antiarrhymics	10.3	16.5	22.3	<0.001	0.646
Amiodarone	5.9	7.0	10.5	0.013	0.511
Diuretics	44.4	39.8	34	0.002	0.168
Vasopressors	0.4	0.2	1.2	0.120	0.926
Antihyperlipidemics	48.1	53.6	48.4	0.102	0.191
NSAIDs	9.8	11.3	13.7	0.119	0.629
COX2 Inhibitors	1.3	1.1	1.2	1.000	0.613
Platelet aggregation inhibitors	13.4	13.1	16.8	0.298	0.066
Other antiplatlets	2.1	2.0	3.1	0.504	0.300
Anti-inflammatory agents	0.1	0.7	0.8	0.008	0.996
Steroids	19.1	17.0	25.4	0.021	0.438
Progestin	0.5	0.0	0.8	0.209	0.369
Estrogen	1.1	1.4	3.1	0.029	0.317
Dronedrone	0.9	3.6	3.5	<0.001	0.948
Ketoconazole	1.5	1.8	0.8	0.572	0.432
Proton pump inhibitor	24.0	24.2	29.3	0.175	0.590

**Fig 2:**
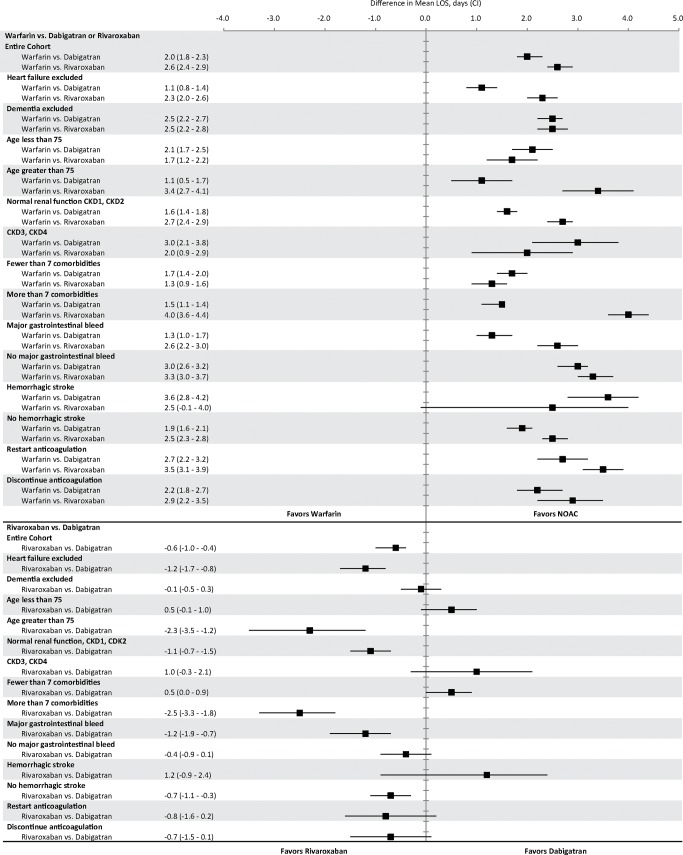
Differences in adjusted mean total length of stay.

We excluded patients if they discontinued anticoagulation (defined as a gap of > 60 days), switched anticoagulants before or after the index admission, died, or lost health plan eligibility prior to admission. We examined pairwise differences in mean total hospital stay among subgroups, which were identified a priori as patients with chronic kidney disease (stage 3 or worse), heart failure, dementia, more than 7 comorbidities, those over 75 years of age, hemorrhagic stroke, major gastrointestinal bleeding, and those who restarted anticoagulation. We defined restarting anticoagulation as a prescription fill of an index anticoagulant within 30 days of discharge from an inpatient stay due to bleeding. **(Fig 2)**

To determine the robustness of our results, we conducted several sensitivity analyses. First, we sought to determined how conditions that might complicate anticoagulation or prolong hospitalization affected our study by repeating the analysis with certain subgroups excluded, including heart failure, chronic kidney disease (stage 3 or worse), 7 or more comorbid conditions, hemorrhagic stroke and, major gastrointestinal bleeding, and those over 75 years of age. Second, we explored an alternative analytic method by adjusting for the propensity scores of dabigatran and rivaroxaban alone. [19]^,^[20]^,^[21]^,^[22] **(S2 Appendix)** And third, because a small but significant difference in the prevalence of cancer and Parkinson’s disease among the three groups persisted after adjustment, we calculated the length of stay and mortality excluding patients with a Parkinson’s or cancer diagnosis. **(S3 Appendix)**

## Results

### Patient characteristics

The cohort consisted of 3,144 patients admitted for bleeding after starting anticoagulation for atrial fibrillation. There were 2,446 warfarin users, 442 dabigatran users, and 256 rivaroxaban users. **(Fig 1)**

Before propensity score weighting, warfarin users were more likely to be older, female, and have heart failure, renal insufficiency, coronary artery disease, major trauma (including fractures), peripheral vascular disease, diabetes mellitus, dementia, and gait abnormalities. After propensity score weighting, there were significant differences between the groups only in the prevalence of cancer (32.1% warfarin, 29.6% dabigatran, 31.6% rivaroxaban, p = 0.01) and Parkinson’s disease (1.4% warfarin, 0.9% dabigatran, 0.4%, p < 0.01). **(Table 1)**

### Total length of hospital stay

Warfarin was associated with significantly longer hospitalizations for bleeding than either dabigatran or rivaroxaban. Mean total length of stay was 7.9 days for warfarin users, 5.8 days for dabigatran users, and 5.2 days for rivaroxaban users. Warfarin users were hospitalized 2.0 days longer (95% CI 1.8–2.3; p < 0.001) than dabigatran users and 2.6 days longer (95% CI 2.4–2.9; p < 0.001) than rivaroxaban users. Dabigatran users were hospitalized 0.6 days longer (95% CI 0.2–1.0; p = 0.001) than rivaroxaban users. **(Table 2)**

**Table 2:** Mean adjusted length of stay, ICU admission, & discontinuation.

**Mean Adjusted Total Length of Hospital Stay**			
	**Warfarin** **(n = 2446)**	**Dabigatran** **(n = 442)**	**Rivaroxaban** **(n = 256)**
**Unadjusted Mean Total Length of Hospital stay, (SD)**	8.9 days (14.14)	6.5 days (7.95)	5.8 days (6.64)
**Adjusted Mean Total Length of Hospital Stay**	7.9 days	5.8 days	5.3 days
**Difference in Adjusted Mean Total Length of Hospital Stay**			
	**LOS, (95% CI)**		
**Warfarin vs. Dabigatran**	2.0 days (1.8–2.3, p < 0.001)		
**Warfarin vs. Rivaroxaban**	2.6 days (2.4–2.9, p < 0.001)		
**Dabigatran vs. Rivaroxaban**	0.6 days (0.2–1.0, p < 0.001)		
**ICU Admission and Length of ICU Stay**			
**Patients with ICU stay**	33%	33%	30%
**Unadjusted Mean ICU Length of Stay (SD)**	9 days (12.08)	6.9 days (6.71)	7.1 days (7.63)
**Adjusted Mean ICU Length of Stay**	10.0 days	7.5 days	8.0 days
**Difference in Adjusted Mean Total ICU Stay among Patients with ICU admission**			
	**LOS, (95% CI)**		
**Warfarin vs. Dabigatran**	3.0 days (1.9–3.9, p < 0.001)		
**Warfarin vs. Rivaroxaban**	2.4 days (0.9–3.7, p = 0.003)		
**Dabigatran vs. Rivaroxaban**	0.6 days (-1.2–2.0, p = 0.490)		
**Discontinuation of Anticoagulation**			
**Discontinuation of anticoagulation,**	37.7%	33.7%	41.4%

### ICU admission and length of stay

There were no significant differences in the proportion of patients admitted to the ICU among the treatment groups, which occurred in 797 (33%) of warfarin users, 146 (33%) of dabigatran users, and 76 (30%) of rivaroxaban users.

Among patients admitted to the ICU, mean length of ICU stay was 10 days for warfarin users, 7.5 days for dabigatran users, and 8.0 days for rivaroxaban users. Warfarin was associated with a 3.0 day longer stay (95% CI 1.9–3.9; p = <0.001) compared to dabigatran and a 2.4 day longer stay (95% CI 0.9–3.7; p = 0.003) compared to rivaroxaban. Mean ICU stay was not significantly different between dabigatran and rivaroxaban. **(Table 2)**

### 30- and 90-day all-cause mortality

All-cause mortality 30 days after discharge was 7.1% among warfarin users, 8.0% among dabigatran users, and 4.6% among rivaroxaban users. After 90 days, the rates were 9.2% among warfarin-users, 9.7% among dabigatran users, and 5.0% among rivaroxaban users. There were no significant differences in relative risk of all-cause 30- or 90-day mortality among the three groups. **(S4 Appendix)**

### Subgroup analyses

Warfarin was associated with longer hospital stays in every subgroup examined. Warfarin is known to be associated with increased risk of intracranial hemorrhage; however, after excluding patients with intracranial hemorrhage, warfarin was still associated with significantly longer hospitalization. Titration of warfarin dose before discharge may prolong hospitalizations; however, warfarin was also associated with longer hospital stay in patients who discontinued anticoagulation and therefore did not require warfarin titration before discharge. In subgroup analyses, there were no consistent differences in length of stay between patients using dabigatran and rivaroxaban. **(Fig 2)**

### Sensitivity analyses

Repeating our analysis with the exclusion of certain subgroups with conditions that might prolong hospitalization or increase mortality did not significantly change outcomes; these subgroups included heart failure, chronic kidney disease (stage 3 or worse), 7 or more comorbid conditions, hemorrhagic stroke and, major gastrointestinal bleeding, and those over 75 years of age.

Outcomes did not significantly change when we applied alternative analytic methods by adjusting for the propensity scores of dabigatran and rivaroxaban alone. **(S2 Appendix)**

Finally, repeating the analysis with the exclusion of patients with either Parkinson’s or cancer diagnosis again suggested the robustness of our findings **(S3 Appendix)**

## Discussion

We found that admission for bleeding during dabigatran or rivaroxaban therapy was associated with shorter total hospital stay and shorter ICU stay compared to admission for bleeding during treatment with warfarin. We found no difference in mortality among the groups but rather a 90-day mortality range of 5.0% to 9.7% that is consistent with those of previously studies,[23]^,^[24]^,^[25]^,^[26] and identifies a vulnerable patient population with post-discharge mortality comparable to that in heart failure.[27]

This study did not confirm our hypothesis that, because anticoagulation with warfarin is more easily reversible, admission for bleeding during warfarin therapy would be associated with shorter hospital stays, fewer ICU admissions, and lower mortality. We considered several hypotheses as to why warfarin might be associated with longer hospital stays.

First, we considered the possibility that warfarin’s known association with increased risk of intracranial hemorrhage was responsible for the prolonged hospitalizations; however, the association of warfarin with prolonged hospitalization persisted after excluding patients with intracranial hemorrhage.

Second, we entertained the possibility that prolonged hospitalization may be due to the need to titrate warfarin before discharge. Alternately the logistical challenges of outpatient warfarin treatment—such as bridging therapy or scheduling in anticoagulation clinic—may have delayed discharge. However, even among patients who discontinued anticoagulation at discharge, warfarin was associated with longer hospitalization. Among patients restarting anticoagulation, warfarin’s association with prolonged hospitalization was stronger, suggesting that the need to titrate warfarin dose and devise appropriate follow up may contribute to the increased medical complexity of warfarin-associated bleeding in some patients.

There are several possibilities why reversibility does not lead to shorter hospitalizations and mortality from bleeding. One explanation may be that reversal of warfarin may not occur rapidly enough to confer benefit compared to the shorter half-life of dabigatran and rivaroxaban. Alternately, clinicians may not recognize the need for reversal of warfarin soon enough to change outcomes, or reversal may be clinically meaningful only in subgroups—such as major trauma—not examined in this study.

Our finding that admission for bleeding during reversible anticoagulation was no shorter than admission for bleeding during irreversible anticoagulation raises the potential that clinicians may be too focused on reversibility rather than on the overall safety profile of a drug. Determining the appropriate role of anticoagulation reversal will become increasingly important given the FDA accelerated approval of idarucizumab—a monoclonal reversal agent for dabigatran—and the publication of a trial showing that andexanet alfa can effect physiologic reversal of rivaroxaban-induced anticoagulation.[28] Andexanet alfa is currently being considered for FDA approval under the “breakthrough therapy” pathway.

The significant risk of mortality after admission for both warfarin- and NOAC-associated bleeding underscores the need for further research into the complexity, severity, and treatment of bleeding during oral anticoagulation. Particular attention should be paid to the newest oral anticoagulants and how associated bleeding is affected by transfusion of blood products and the use of reversal agents.

## Study limitations

This study examines outcomes after bleeding has occurred, not the risk of bleeding during anticoagulation with warfarin or NOACs. This study is also subject to the well-known limitations of medical claims analysis, particularly a lack of granularity of in some aspects of the data. For example, identification of a hospitalization for hemorrhage relied upon the use of one of the relevant ICD-9 codes in the insurance claim. Similarly it was not possible to determine if certain comorbidities arose before or during the hospitalization for hemorrhage. However, while the absence of granularity is regrettable, these limitations should affect all three treatment groups in a non-differential manner. Other limitations include the observational design and an inability to capture fatal bleeding before admission.

Importantly, warfarin users were significantly older and sicker, and despite careful adjustments, this disadvantage may have accounted for the observed differences in outcomes. However, the many sensitivity analyses we undertook demonstrated robust results. Moreover, the marked differences in length of stay after statistical adjustment for a large number of variables make it unlikely that the observed differences in outcomes are purely the result of residual confounding. Finally, our results are consistent with clinical trial data. An analysis of the RE-LY trial found that patients admitted for major bleeding while on dabigatran had shorter ICU stays and no difference in mortality when compared to patients admitted for major bleeding on warfarin.[29] An analysis of the ROCKET-AF trial found that patients who experienced major bleeding during rivaroxaban therapy had no difference in all-cause mortality compared to those with major bleeding on warfarin.[30]

Notably, our study found a lower all-cause 30-day mortality than that previously described in the trial data. An analysis of ARISTOTLE trial found no difference in 30 day all-cause mortality for patients on either apixaban or warfarin who had major bleeding events.[31] However, that study noted a 30 day mortality of 14.9%, which contrasts to far lower 4.6–8.0% noted in our study. This discrepancy is likely the result of two factors. First, ARISTOTLE was a multinational trial in which Russia, Argentina, Ukraine, and China were among the top six enrolling countries; therefore, it is expected that our study of insured Americans would have a lower 30 day all-cause mortality. Second, trial registries may more completely capture mortality than retrospective interrogation of discharge status and linkage to the Social Security Administration Death Master File.

It must be noted that the Death Master File made a change in how it disclosed its mortality in November 2011that may have resulted in underreporting of mortality[32]. Because our study started on 1 November 2010 with dabigatran’s entry into the market, and because rivaroxaban was not FDA approved until 4 November 2011, this introduces the possibility for a differential capture of mortality. Therefore, we conducted a sensitivity analysis on the mortality findings by restricting data from 1 November 2011 to 31 March 2014 and found no significant change. (**S5 Appendix**)

It is possible that the differences in length of stay may be caused by differential admission and treatment of bleeding during oral anticoagulation. We were unable to examine clinician decision-making at the time of admission or the use of blood products and reversal agents. It is possible that clinicians were more wary of dabigatran- and rivaroxaban-associated bleeding and therefore had a lower threshold for admission; however, equal proportions of each treatment group were admitted to the ICU, suggesting comparability. While we were unable to compare blood product transfusions, analysis of RE-LY data found that patients admitted with major bleeding during dabigatran therapy receive more transfusions of red blood cells than those admitted for major bleeding during warfarin therapy.[29]

## Conclusions

Rivaroxaban and dabigatran were associated with shorter hospitalizations; however, there were no differences in 30- and 90-day mortality. These findings suggest bleeding associated with the newer agents is not more dangerous than bleeding associated with warfarin.

## Supporting information

S1 Appendix Definition of covariates. (TIF)

S2 Appendix Alternative analytic method of adjusting patient characteristics. (TIF)

S3 Appendix Effect of cancer & parkinson’s on mean length of stay & all-cause mortality. (TIF)

S4 Appendix All-cause 30- & 90-day mortality. (TIF)

S5 Appendix All-Cause 30 & 90-day mortality following hospitalization for bleeding after November 1, 2011. (TIF)
