# Supplementary figures and images for: Length of hospitalization and mortality for bleeding during treatment with warfarin, dabigatran, or rivaroxaban
Source: PLoS One. 2018 Mar 28;13(3):e0193912. doi: 10.1371/journal.pone.0193912 (PMC5874024; doi:10.1371/journal.pone.0193912)

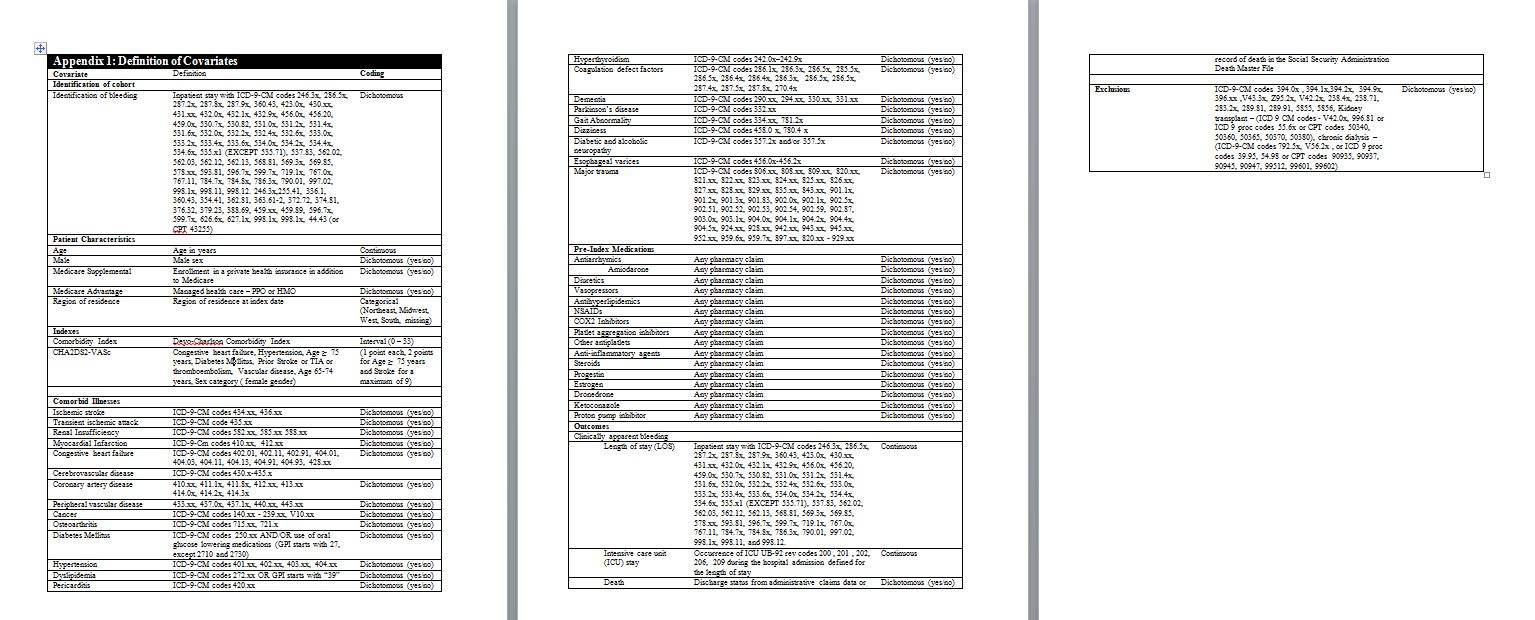

Supplement: S1 Appendix — (TIF) [file pone.0193912.s001.tif]

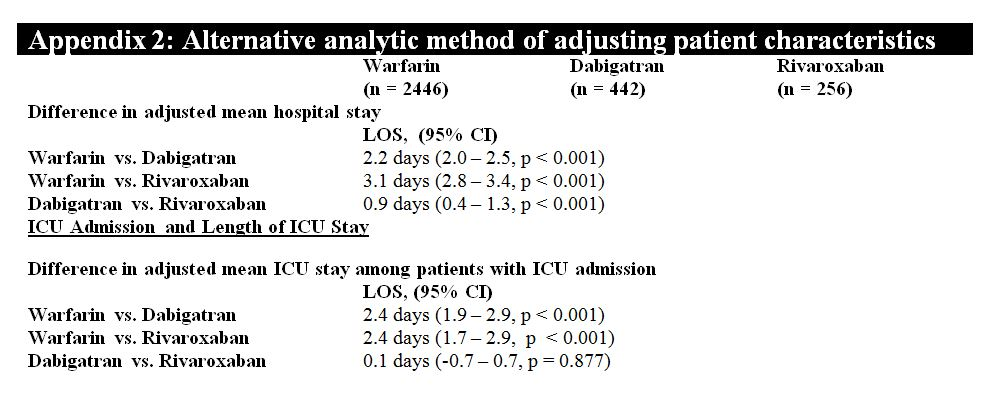

Supplement: S2 Appendix — (TIF) [file pone.0193912.s002.tif]

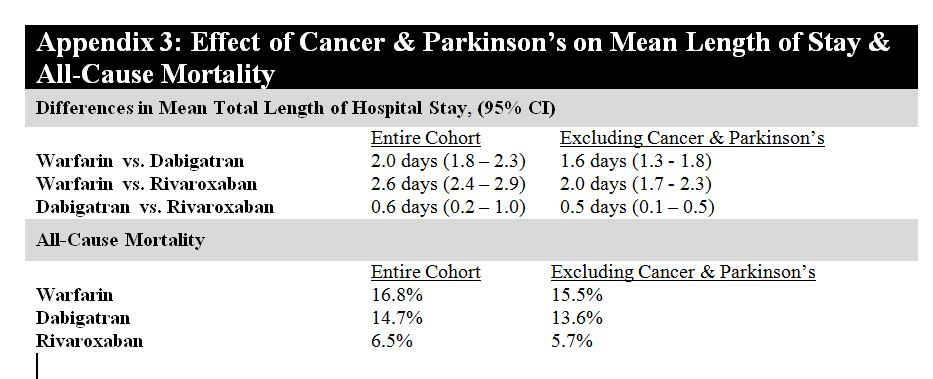

Supplement: S3 Appendix — (TIF) [file pone.0193912.s003.tif]

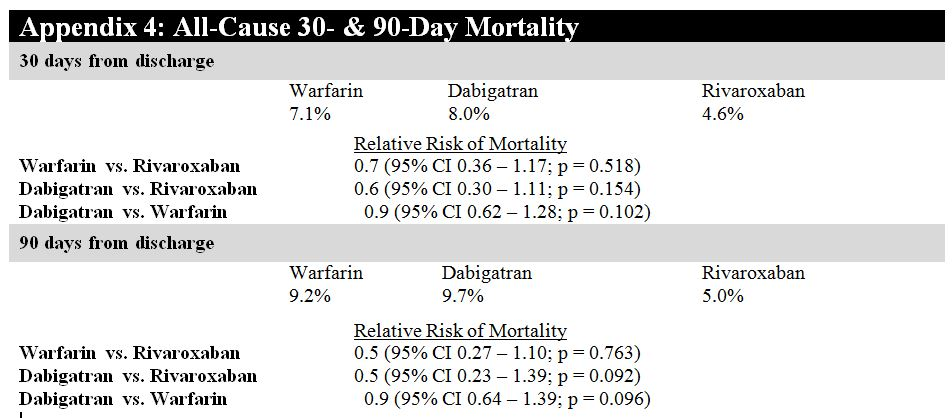

Supplement: S4 Appendix — (TIF) [file pone.0193912.s004.tif]

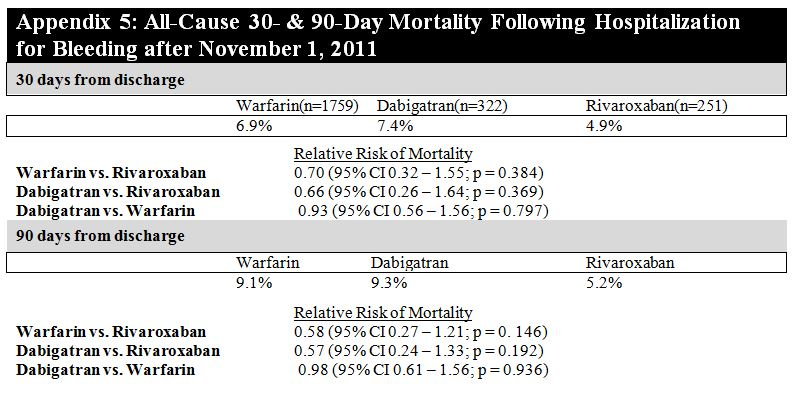

Supplement: S5 Appendix — (TIF) [file pone.0193912.s005.tif]
